# Prevalence of night eating syndrome and associated factors among students at Kolej MARA Kuala Nerang: A cross-sectional study

**DOI:** 10.51866/oa.977

**Published:** 2026-09-13

**Authors:** Sook Ching Chan, Sharif Zaidi Nurhannah, Magdalena Alphonso Ribka

**Affiliations:** 1 Community Based Department, Faculty of Medicine, Royal College of Medicine Perak, Universiti Kuala Lumpur, 3 Jalan Greentown, Ipoh, Perak, Malaysia.; 2 Hospital Sultan Abdul Halim, 225 Bandar Amanjaya, Sungai Petani, Kedah, Malaysia.; 3 Hospital Sibu, Batu 5 1/2, Jalan Ulu Oya, Pekan Sibu, Sibu, Sarawak, Malaysia.

**Keywords:** Night eating syndrome, Students, College, Feeding and eating disorders

## Abstract

**Introduction:**

Night eating syndrome (NES) is an eating disorder characterised by recurrent episodes of night eating, morning anorexia and insomnia. This study aimed to determine the prevalence and association of NES with depression, anxiety, stress, sleep quality and eating disorders among students in Kolej MARA Kuala Nerang (KMKN).

**Methods:**

A cross-sectional study was conducted on 426 students in KMKN. The calculated minimum sample size was 119. A universal sampling method was used, and data on sociodemographic characteristics, NES, depression, anxiety, stress, sleep quality and eating disorders were collected through online self-reported validated questionnaires including the Night Eating Questionnaire; Depression, Anxiety and Stress Scale-21; Pittsburgh Sleep Quality Index; and Eating Attitudes Test- 26. Data were analysed using IBM SPSS version 22. The chi-square test was used to evaluate the association of NES with sociodemographic characteristics and other factors (P<0.05).

**Results:**

A total of 275 students responded (men: 55.6%, women: 44.4%), with 50.9% studying at the Foundation of Science and 49.1% at the Foundation of Science and Technology (FIST). The majority had mild-to-severe depression (56.4%) and anxiety (65.5%); 26.2% were stressed, and 10.2% engaged in NES. NES was not significantly associated with sex, age, monthly pocket money or body mass index. A significant number of students with NES were from the FIST (P=0.036). The factors significantly related to NES were depression (P=0.003), anxiety (P=0.021), stress (P<0.001), poor sleep quality (P=0.001) and risk of eating disorders (P=0.003).

**Conclusion:**

NES should be screened among at-risk college students to ensure they receive appropriate treatment.

## Introduction

Eating disorders are a common chronic illness among adolescents (aged 10–19 years), after obesity and bronchial asthma, with a peak onset^1,2^ at 14–19 years of age.^3^ Night eating syndrome (NES) was defined by Stunkard et al. as a collection of behavioural problems that include morning anorexia, evening hyperphagia with 25% of the total energy intake after 7 p.m. and insomnia.^3^ Allison and colleagues had proposed a new definition of NES as a circadian delay in the pattern of food intake.^4^ NES is categorised as another specified feeding or eating disorder in the Diagnostic and Statistical Manual of Mental Disorders, 5^th^ edition (DSM-5). In the DSM-5, NES is defined as recurrent episodes of night eating, manifested by eating after awakening from sleep or by excessive consumption after the evening meal. This syndrome also includes the awareness and ability to recall the night eating with significant distress and/or impairment of the functioning of the individual.^5^ A young adult who is transitioning into university life has a high tendency to adopt a poor eating habit due to stress.^6^ A study conducted in three provinces in China showed that 2.8% of Chinese university students engaged in NES.^6^ In Malaysia, a published study on public university students indicated that 12.2% engaged in NES.^7^ Gan et al. conducted the study on 270 participants from a public university in Selangor state. Students were from three faculties (arts, science and technical), among whom 12.2% engaged in NES (Night Eating Questionnaire [NEQ] cutoff score of >25).^7^

The prevalence of NES in the general population was found to range from 1.1% to 1.5% in a German study.^8^ Four significant associated factors were determined, which included male sex, technical courses, a mild stress level and poor sleep quality.^7^

Depression, anxiety and stress have also been identified as associated factors of NES. Gan and colleagues reported that only stress had a strong association with NES.^7^ A strong association between NES and depression, anxiety and stress was found in two other studies.^8,9^ Three main reasons identified among students who developed depression, anxiety and stress were academic performance, pressure to succeed and post-graduation plans.^10^

Another associated factor of NES is poor quality of sleep. Gan et al. and Cleator et al. both reported an association between poor sleep quality and NES.^7,11^ There was a weak relationship between the NEQ score and sleep latency and duration; thus, the sleep—wake cycle was relatively undisturbed in NES.

Most spectra of eating disorders, especially anorexia nervosa, bulimia nervosa and binge eating, share the same characteristics, which include a fear of fatness and the pathological preoccupation with food, weight and body shape.^2^ The Eating Attitudes Test is used to determine the presence of eating disorders, mainly anorexia nervosa and bulimia nervosa.^12^ Both Gan et al. and Cengiz et al. found no association between NES and other eating disorders.^7,13^

Currently, there are few studies conducted on NES in Malaysia. NES can cause obesity and metabolic disorders later in life if untreated.^14^ This study aimed to determine the prevalence of NES among students in Kolej MARA Kuala Nerang (KMKN) and its association with sociodemographic characteristics, depression, anxiety, stress, sleep quality and disordered eating behaviour. This study could contribute to future interventions for this condition and its complications.

## Methods

A cross-sectional study was conducted in KMKN to determine the prevalence and associated factors of NES among students. This study was a medical student’s research project under the supervision of a lecturer with prior approval from the Director of KMKN. Ethical approval was granted by the Medical Ethics Committee of Universiti Kuala Lumpur Royal College of Medicine Perak (Ref. No.: UniKL RCMP/MREC/2019-2020/SRP-120). Respondents were assured that none of their identification or personal information would be disclosed. An online consent form was obtained before data collection.

With a prevalence of NES of 12.2%, a confidence level of 95% and a total population of 426 students in KMKN, the required sample size was calculated as 119 using OpenEpi. The original planned sample size was at least 130, but since the total population and the sample size were small, a universal sampling method was used to factor in potential dropouts or incomplete/ missing data.

Students’ sociodemographic characteristics, NES, depression, anxiety, stress, sleep quality and eating disorder data were collected through an online self-reported questionnaire. The questionnaire was divided into three segments: sociodemographic data, the NEQ score and associated factors. The sociodemographic and anthropometric data gathered included sex, age, field of study, height, weight, body mass index (BMI) and monthly pocket money.

NES was assessed using the NEQ.^15,16^ Sixteen questions assess the behavioural and psychological syndrome of NES.^7^ The components that evaluate NES are the amount of daily food intake during evening or nighttime (one item), the presence of nocturnal eating (two items), the awareness and ability to recall the nocturnal eating (one item), the lack of desire to eat breakfast (two items), the craving to eat during nighttime or after onset of sleep (two items), insomnia (four items), mood (two items) and whether the syndrome affects their life (two items).^16^ In this study, one question was added to assess the average evening meal. This questionnaire has been validated among university students in a previous study.^7^

Depression, anxiety and stress were evaluated using the Depression, Anxiety and Stress Scale-21 (DASS-21). Each component has a total of seven items that assess the psychological and emotional states of respondents.^7^ The DASS-21 is a valid instrument used to assess respondents’ levels of depression, anxiety and stress.^7^ Conversely, the Pittsburgh Sleep Quality Index (PSQI) was used to assess the quality of sleep over a 1-month duration. It has 10 items, including subjective sleep quality, sleep latency, sleep duration, habitual sleep efficiency, sleep disturbance, use of medication and daytime dysfunction. Among university students, the PSQI is a reliable and validated instrument.^7^ Disordered eating behaviour was evaluated using the Eating Attitudes Test-26 (EAT-26). This test has three subscales, which include dieting, bulimia and food preoccupation. The EAT-26 has been validated and is commonly used among Malaysian university students.^7^

All data were collected online through Google Forms and tabulated in Microsoft Excel. For data analysis, sociodemographic data underwent descriptive analysis. In the NEQ, 14 out of the 17 items are graded using a Likert scale as follows: 0 *(not at all),* 1 *(a little),* 2 *(somewhat),* 3 *(moderately)* and 4 *(very).* The other three questions (items 15–17) are not given a score. Three items are reversely scaled (items 1, 4 and 14). The scores for items 1–14 are summed up. Thereafter, the total score is categorised into with and without NES with a cutoff point of ≥25.

The DASS-21 items are scored using a Likert scale as follows: 0 *(not applied to me at all),* 1 *(applied to me to some degree or some of the time),* 2 *(applied to me to a considerable degree or a good part of the time)* and 3 *(applied to me very much or most of the time).* All item scores are summed up separately based on each component: depression, anxiety and stress. The total scores are then classified into different levels as follows: normal (0–14), mild (15–18), moderate (19–25) and severe (26–33).

The PSQI is scored using a Likert scale as follows: 0 *(not during the past month*), 1 *(less than once a week*), 2 *(once or twice a week*) and 3 *(three or more times a week*). Item 9 is scaled differently as follows: 0 *(very good*), 1 *(fairly good*), 2 *(fairly bad*) and 3 *(very bad*). The scores for seven components are summed up according to the global PSQI standardised questionnaire.^17^ The total score is classified into good and poor sleep quality with a cutoff point of >5.

The EAT-26 items are scored using the following Likert scale: 0 *(never),* 0 *(rarely),* 0 *(sometimes),* 1 *(often),* 2 *(usually)* and 3 *(always).* Item 26 is reversely scaled. All item scores are summed up, and data are classified into normal and at risk of having an eating disorder with a cutoff point of ≥20.

Statistical analysis was conducted using IBM Corp. IBM SPSS Statistics for Windows, Version 22.0. Armonk, NY: IBM Corp; 2013. The findings of the univariate analysis for continuous data were presented as means and standard deviations and those for categorical data as frequencies and percentages. The association of NES with the sociodemographic characteristics and other factors (depression, anxiety, stress, sleep quality and eating disorders) was examined using the chi-square test.

## Results

A total of 275 students responded to the questionnaire, among whom 55.6% were men, and 44.4% were women. Approximately 50.9% were from the Foundation of Science (FSc) and 49.1% from the Foundation of Science and Technology (FIST). The majority of the respondents (57.5%) received RM 100 or less per month. For BMI, 50.2% had a normal BMI of 19-24.9 kg/m^2^; conversely, 18.2% were underweight (BMI of <19 kg/m^2^); 22.5%, overweight (BMI of 25-29.9 kg/m2); and 9.1%, obese (BMI of ≥30 kg/m^2^) (Table 1).

**Table 1:** Sociodemographic characteristics of the respondents (N=275).

Sociodemographic characteristic	n (%)
Sex	
Male	153 (55.6)
Female	122 (44.4)
Age	
18 years	21 (7.6)
19 years	254 (92.4)
Field of study	
Foundation of Science and Technology	135 (49.1)
Foundation of Science	140 (50.9)
Monthly pocket money	
≤RM 100	158 (57.5)
>RM 100	117 (42.5)
BMI^*^	
Underweight (<19 kg/m^2^)	50 (18.2)
Normal (19–24.9 kg/m^2^)	138 (50.2)
Overweight (25–29.9 kg/m^2^)	62 (22.5)
Obese (≥30 kg/m^2^)	25 (9.1)

* Body Mass Index. World Health Organization 2019

With the total NEQ score categorised into with and without NES using a cutoff point of >25, 28 (10.2%) participants were found to be engaged in NES, while most participants (n=247, 89.8%) were not engaged in NES.

The majority of the participants (56.4%) had depression: mild (21.1%), moderate (21.5%) and severe (13.8%). One hundred and eighty participants (65.5%) experienced anxiety, with 27.6% reporting severe anxiety; 26.9%, moderate anxiety; and 10.9%, mild anxiety. Most had poor sleep quality (65.1%). The majority had a normal eating attitude (81.5%) (Table 2).

**Table 2:** Psychological and behavioural characteristics of the respondents (N=275).

Psychological and behavioural characteristics	n (%)
Depression	
Normal	120 (43.6)
Depression (total)	155 (56.4)
Mild	58 (21.1)
Moderate	59 (21.5)
Severe	38 (13.8)
Anxiety	
Normal	95 (34.5)
Anxiety (total)	180 (65.5)
Mild	30 (10.9)
Moderate	74 (26.9)
Severe	76 (27.6)
Stress	
Normal	203 (73.8)
Stress (total)	72 (26.2)
Mild	27 (9.8)
Moderate	27 (9.8)
Severe	18 (6.5)
Sleep quality	
Good	96 (34.9)
Poor	179 (65.1)
Eating disorder	
Normal	224 (81.5)
At risk	51 (18.5)

Only the field of study was found to be significantly associated with NES (Table 3). Sex, age, monthly pocket money and BMI were not significantly associated with NES.

**Table 3:** Association between NES and sociodemographic characteristics among the respondents (N=275).

Sociodemographic characteristic (n)	Without NES		With NES		X^2^	P
	n	%^*^	n	%^*^		
Sex					0.029	0.866
Male (153)	137	89.5	16	10.5		
Female (122)	110	90.2	12	9.8		
Age					0.419	0.518
18 years (21)	18	85.7	3	14.3		
19 years (254)	229	90.2	25	9.8		
Field of study					4.393	0.036
Foundation of Science and Technology (135)	116	85.9	19/135	14.1		
Foundation of Science (140)	131	93.6	9/140	6.4		
Monthly pocket money					1.550	0.231
≤RM 100.00 (158)	145	91.8	13/158	8.2		
>RM 100.00 (117)	102	87.2	15/117	12.8		
BMI					1.032	0.794
Underweight (50)	43	86	7	14		
Normal (138)	125	90.6	13	9.4		
Overweight (62)	56	90.3	6	9.7		
Obese (25)	23	92	2	8		

* The denominator is n in the first column. NES: night eating syndrome.

Depression, anxiety, stress, sleep quality and eating disorders were significantly associated with NES (Table 4).

**Table 4:** Association between NES and psychological and behavioural characteristics among the respondents (N=275).

Associated factor (n)	Without NES		With NES		X^2^	P
	n	%^*^	n	%^*^		
Depression						
Normal (120)	115	95.8	5	4.2		
Depressed (total) (155)	132	85.2	23	14.8	8.4234	0.003
Mild (58)	54	93.1	4	6.9	20.801	<0.001
Moderate (59)	51	86.4	8	13.6		
Severe (38)	27	71.1	11	28.9		
Anxiety						
Normal (95)	91	95.8	4	4.2		
Anxiety (total) (180)	156	86.7	24	13.3	5.6589	0.017
Mild (30)	28	93.3	2	6.7	9.783	0.021
Moderate (74)	66	89.2	8	10.8		
Severe (76)	62	81.6	14	18.4		
Stress						
Normal (203)	190	93.6	13	6.4		
Stressed (total) (72)	57	79.1	15	20.8	12.1005	<0.001
Mild (27)	25	92.6	2	7.4	20.656	<0.001
Moderate (27)	19	70.4	8	29.6		
Severe (18)	13	72.2	5	27.8		
Sleep quality					10.577	0.001
Good (96) Poor (179)	94 153	97.9 85.5	2 26	2.1 14.5		
Eating disorder					8.877	0.003
Normal (224) At risk (51)	207 40	91.2 78.4	17 11	8.8 21.6		

* The denominator is n in the first column. NES: night eating syndrome.

## Discussion

The prevalence of NES in this study was 10.2%. This is lower than the prevalence reported by Gan and colleagues (12.2%) despite the almost equal total number of students (N=275 and N=270, respectively).^7^

In their study conducted among 1636 students from 10 US universities on the prevalence and clinical significance of NES, Runfola et al. showed a prevalence of 4.2%.^16^ A meta-analysis of the prevalence of NES associated with psychological disorders (e.g. sleep disturbance, depression and anxiety) among university students from 13 studies worldwide showed a prevalence ranging from 1.2% to 35.4%.^18^ The majority of NES tests showed that it was more common among college students than in the general population.^18,19^ The reasons for this disparity were that college students had higher stress levels and more sleeping difficulties.^9^

Among all sociodemographic characteristics, only the field of study was significantly associated with NES. Gan and colleagues reported a similar finding in that the technical course was significantly associated with NES.^7^ The difference between the FSc and the FIST is that each course has different types of science subjects. The FSc studies biology, while the FIST studies physics. Korinth et al. reported that students with nutritional knowledge made better nutritional choices and prevented unhealthy eating habits.^20^

Depression, anxiety and stress were significantly associated with NES. In their study, Borges et al. found similar results.^8^ The association of depression, anxiety and stress with NES has been consistent across the literature.^21,22^ Another study revealed that individuals who engaged in NES presented with more depressive symptoms and stress than healthy controls.^23^ Students who were experiencing a highly stressful event and developed a less adaptive coping ability had a tendency to engage in night eating.^22^ The study by Beiter et al. found three main reasons in students who developed depression, anxiety and stress: academic performance, pressure to succeed and postgraduation plans.^10^

Poor sleep quality has also been identified to be significantly associated with NES.^7,21^ A study discovered that individuals who engaged in NES with evening overconsumption had less sleep difficulty and lifestyle impairment than individuals with night ingestion.^21^ Nolan and Geliebter suggested that stressed individuals who experience low mood prior to bedtime have a greater tendency to wake up at night and are thus more likely to engage in emotional eating to reduce the negative feeling or thought.^21,22^ According to Gan and colleagues, cohort studies should be conducted to determine whether sleep difficulties precede or cause NES.^7^

In this study, another associated factor was other eating disorders (P=0.003). Studies reporting the association between NES and other eating disorders are limited, but NES could be differentiated from other conditions by the nature of ingestion and clinical symptoms.^9^

One limitation of this study is that it was conducted in one college. Given the cross-sectional study design, causal inferences regarding the associations between NES and risk factors cannot be made. Another limitation is the lack of adjustment for confounding, particularly for depression, anxiety, stress, sleep quality, eating disorder risk and field of study. A cohort study design should be applied to determine the cause of the association. Moreover, there might be recall or information bias, since the data were collected online, and the students needed to recall information prior to answering the questions.

## Conclusion

This study showed that the prevalence of NES among the participants was 10.2%. Significantly more students from the FIST course had NES than those from the FSc course. Psychological and behavioural characteristics such as depression, anxiety, stress and sleep quality were significantly associated with NES.

Recent reviews indicate that NES is a complex disorder with multifactorial aetiology,^24,25^ usually appearing in late adolescence/early adulthood, which corresponds to the period of college/ university attendance. It appears to persist with relapse and remissions related to stress. If NES is untreated, several health issues are found to be related, such as obesity, high cholesterol, diabetes and hypertension.^26^ NES is also associated with depression, anxiety and stress. NES should be screened in the early stage, and treatment should be offered to prevent these long-term issues and address associated factors early.

Therefore, the authors suggest increasing the awareness of NES among college/university students (e.g. via health campaigns) and encourage further assessment and support for students with night eating symptoms and related psychological or sleep concerns.

## Data sharing statement

Our raw data are available only upon request.

**How does this paper make a difference in general practice?**

- This study shows that college students are at risk of developing night eating syndrome and experiencing depression, anxiety, stress, poor sleep and eating disorders.
- Primary care doctors should be aware of these factors and screen at-risk patients.
